# Perception of the operation theater learning environment and related factors among anesthesia students in Ethiopian higher education teaching hospitals: a multicenter cross-sectional study

**DOI:** 10.1186/s12909-024-05320-6

**Published:** 2024-03-19

**Authors:** Habtemariam Wubshet, Abatneh Feleke Agegnehu, Misganaw Mengie Workie, Yonas Addisu

**Affiliations:** 1 Department of Anesthesia, College of Medicine and Health Sciences, University of Gondar, Gondar, Ethiopia; 2 Department of Anesthesia, College of Medicine and Health Sciences, Debre Berhan University, Debre Berhan, Ethiopia

**Keywords:** Perception, Operation theater, Learning environment, Anesthesia

## Abstract

**Introduction:**

Operation theater learning involves three key elements: clinical work, learning, and the environment. There is little evidence regarding the operating theatre learning environment for anesthesia trainees. Identifying the overall perception of the operation theater learning environment helps to establish an efficient operation theater learning environment and produce competent anesthesia professionals.

**Objective:**

The aim of this study was to assess the perceptions of the operating theater learning environment and associated factors among undergraduate anesthesia students in Ethiopian higher education teaching hospitals from April to May 2023.

**Methods:**

A multicenter cross-sectional study was conducted on 313 undergraduate anesthesia students who began operation room clinical practice at 13 higher education teaching hospitals. The data were entered into EpiData version 4.6. A generalized ordered logistic regression model was used to analyze and identify factors associated with the operating theater learning environment using STATA software version MP17.

**Results:**

The findings of this study revealed that 45.05%,26.52%), 23% and 5.43% of the participants reported having desirable, moderately desirable, very desirable and undesirable perceptions of the operating theater learning environment, respectively, from highest to lowest. Preoperative discussion (AOR = 4.98 CI = 1.3–18.8), lack of teaching facilities (AOR = 0.16 CI = 0.03–0.75), noise from played music (AOR = 0.22 CI = 0.07–0.63), absence of tutors (AOR = 0.03 CI = 0.01–0.22), respect for students (AOR = 3.44 CI = 1.6–7.2), roll modeling for students (AOR = 3.23 CI = 1.5–6.8) and strict supervision of students (AOR = 0.24 CI = 0.07–0.88) were significantly associated with perceptions of the operation theater learning environment, with 95% CIs.

**Conclusion:**

No study participant agreed that the operation theater learning environment in operation theatres was very undesirable. A lack of teaching facilities at the OR, a lack of tutors from the OR, noise from played music in the OR, a tutor respecting their student, a tutor role model for their student, a preoperative discussion with a tutor and strict supervision of the student are strongly associated with the operation theatre learning environment.

**Supplementary Information:**

The online version contains supplementary material available at 10.1186/s12909-024-05320-6.

## Introduction

Modern medicine would be unthinkable without the profession of anesthesia. On October 16, 1846, the surgeon John Warren and dentist William T.G. Morton performed the first operation under a primitive form of anesthesia at Massachusetts General Hospital in Boston. Among medical specialties, anesthesia is unique in that it requires extensive knowledge in three major areas of learning: cognitive (knowledge), psychomotor (procedural skills), and affective (nontechnical skills) [1].

Anesthesiologists have been among the pioneers of patient safety [2]. Anesthesiology is a branch of medicine that addresses the treatment of surgical patients before, during and after surgery, as well as pain management, and is involved in the treatment of patients who require intensive care [3]. Students should be able to practice their theoretical knowledge at clinical practicum sites, develop their clinical thinking and clinical skills, and adapt to professional situations through clinical practice [4].

In operation theater learning environments, theory is translated into practice, and it is a clinical classroom that includes three key elements: clinical work, learning, and environment [5]. The majority of anesthesia educators believe that the most typical form of anesthesia education is teaching in the operating room (OR) [6, 7]. The OR is a complex environment in which a variety of factors, such as advanced equipment and the sensitivity of clinical situations, create a unique and engaging learning experience; however, if not done properly, teaching can occasionally lead to cognitive overload for both the instructor and the learner [6, 8].

There are Lyon’s three key categories of obstacles for operation theater learning (OTL): physical setting, the learning task in that setting, and the difficulty of managing social relationships in theaters [9]. The operating room is a special setting with its own culture, procedures, and interdisciplinary team. Many of whom have been working together for a very long time [10]. Quality has a special meaning in clinical education, so clinical education can be viewed as a learning activity in a clinical setting involving the clinical instructor and the student [11]. The Accreditation Council for Graduate Medical Education (ACGME) developed 6 core competencies that can be taught effectively while teaching in the OR [12]. These include patient care, clinical knowledge, practice-based learning improvement, role models, communication skills, professionalism and system-based practice [13].

The operating theater learning environment is thought to influence learner behavior and predict learner competency in clinical practice. An assessment of the operating theater learning environment is a technique for evaluating the quality of educational programs [14]. The operation theater learning environment (OTLE) is not always optimal for learning because of clinical productivity expectations and a lack of support from supervisors.

Operation room (OR)-based student learning has traditionally been suboptimal. Learning in operating rooms and similar settings can be difficult. Even in teaching hospitals, patient safety must remain the top priority for all staff members, and in this respect, learning plays a secondary role for instructors [15].

The effectiveness of clinical teaching and learning can be influenced by several factors. A faculty member conducts clinical instruction within the framework of a curriculum designed and delivered in response to professional, societal, environmental, and educational expectations and demands using the available human, intellectual, material, and financial resources of the curriculum [16]. The provision of the instructor, proper management, and availability of opportunities for learners are just some of the aspects that determine the success or failure of theory-practice integration [17].

Effective supervision, a sufficient number of tutors, and clinical instructors are facilitating factors, and a lack of self-confidence, absenteeism, inadequate supervision, and a lack of resources are obstacles to effective clinical learning [18]. Effective teaching efforts in the OR may be hindered by considerable problems that may be present at the same time, such as medical-legal issues and production pressure, these difficulties, teaching anesthesiology in the operating room will likely continue to be the primary educational setting for their specialty [6]. Some studies have outlined a few of the advantages that students look for in an excellent instructor, including excellence in clinical practice, demonstration of clinical excellence, active engagement of learners, positive attitude toward teaching, ability to create a positive overall environment, role models of professionalism and overall concern for the learner [19]. Before their OR rotation, students who receive formal training in virtual environments do better than those who do not, and undergraduates can practice in a safe environment using simulations before their theatre placement [20]. The OR experience can be greatly improved with the use of didactic lectures, web conferences, online seminars, pretheatre workshops, virtual training, and simulated operation suites (SOS) [21]. Although every anesthesiologist learns his or her craft in the operating room, the majority of anesthesia educators are not trained in how to teach effectively in this environment [6]. The physical environment may make it difficult to teach clinical skills due to loudness, a lack of privacy for giving feedback, or a lack of space for training [22].

In the operating room, learning interactions between the clinical instructor and the student are often brief, unplanned, spontaneous and opportunistic. Although most operating room teachers have extensive teaching experience in their setting, only a few are able to enhance and maximize opportunities and learning obstacles [23]. Clinical instructors must become proficient in the techniques and strategies that will enhance teaching and learning in operating rooms. Understanding learning methodologies and principles improves instructors’ capacity to provide successful instruction and fosters gratifying faculty-learner engagement [24, 25]. Learning settings are among the crucial aspects of learning, along with the skills and traits of clinical teachers [26].

Operating room learning is an important phase in anesthesia students’ training; however, it remains unstructured [27]. students have reported inadequate organizational support for OR-based learning [24]. When learning objectives are futile, they hinder learning and cause dissatisfaction and confusion [22].

Raindrop and colleagues reported that 47% of the students were uncertain learning objectives when asked about them [22]. To achieve this goal, the importance of a comprehensive OR orientation session is pivotal. It is imperative to emphasize senior faculty members’ active involvement in mentoring and supervising students if students are to comprehend the significance of their theatre placement. Teamwork and a sense of feeling socially included are powerful determinants that positively influence learning. The friendliness and approachability of staff are the most important factors, and they are reported to affect learning [28]. A lack of orientation about the OR environment induces stress and confusion [27, 29].

Numerous studies have investigated the factors influencing the operating theater learning environment outside of the operating room; however, there is little evidence regarding the operating theatre learning environment for anesthesia trainees [30]. It is essential to determine what the operating room learning environment should look like to increase students’ enthusiasm, strength and empowerment through the use of proper educational strategies and teaching methods to boost students’ interest in anesthesia clinical practice.

To create standardized OTLEs in the future, at least a baseline study is needed, and exploration of Ethiopian anesthesia students’ operation theater learning environment will continue to be an important research focus. The aim of this study was to evaluate the learning environment in operating theaters and its associated factors among undergraduate anesthesia students in Ethiopian higher education teaching hospitals. Specifically, our objectives are to assess the learning environment in operating theatres among undergraduate anesthesia students in Ethiopian higher education teaching hospitals and to identify the factors influencing the operating theatre learning environment. The focus of the study is on evaluating the learning environment in operating theaters for undergraduate anesthesia students, with the aim of enhancing educational quality in this field. By identifying factors influencing the learning environment, such as teaching methods and resources, this study seeks to inform improvements in teaching practices and student experiences. This can lead to increased satisfaction, motivation, and engagement among students undergoing anesthesia training. Insights from the study can also inform targeted interventions to optimize learning outcomes and competency development. Furthermore, the study serves as a baseline for ongoing evaluation and improvement of the learning environment, facilitating continuous enhancement of educational quality. Additionally, this research contributes to the existing knowledge on anesthesia education and learning environments, particularly in Ethiopian higher education teaching hospitals, benefiting future research and facilitating cross-cultural comparisons.

## Methods and materials

A multicenter cross-sectional study design was employed to evaluate the perceptions of the operation theater learning environment and related factors among undergraduate anesthesia students in Ethiopian higher education teaching hospitals. The study was conducted from April to May 2023. The study included all undergraduate anesthesia students in Ethiopian higher education teaching hospitals who commenced clinical practice in the operating theatre. Data collection utilized a survey method, with a self-administered questionnaire distributed to each university. A representative from each university facilitated the distribution of the questionnaire to every student, ensuring the inclusion of all students from each university. The convenience sampling method was employed in this study. To perform the study, the University of Gondar School of Medicine provided ethical approval. Participants had the freedom to fill or ignore participation in this study. The names of participants were removed from questionnaires to ensure confidentiality. Due to its practicality, all students were incorporated into this study. However, upon completion, data from seven participants were found to be incomplete and were consequently excluded from the study. To ensure data quality, we conducted pretesting of the data collection tool.

### Sample size determination

In Ethiopia, 27 universities offer undergraduate anesthesia training, but only 13 of them have teaching hospitals. Our research focused solely on universities with teaching hospitals. Initially, 328 students started the anesthesia module course and clinical practice in the operating room, and we were able to include all of them in our study. However, data from seven participants were incomplete, leading to their exclusion. As a result, the final sample size comprised 313 students.

The 13 universities with their corresponding numbers of students are as follows: 57 students at Addis Ababa University, 10 students at the University of Gondar, 20 students at Jimma University, 23 students at Hawassa University, and 30 students at Bahir Dar University. Dilla University − 18 students, Wolaita Sodo University − 19 students, Menelik II College − 39 students, Debre Birhan University − 22 students, Adama Health Science College − 13 students, Wachemo University − 33 students, Arsi University − 32 students, Bule hora University − 12 students.

#### Dependent variables

Perception to Operation Theater learning environment.

**The dependent** variables included sex, clinical instructor factor, emotional factors, socio environmental factors and organizational factors.

### Operational definition

The perception of the operating theater learning environment is categorized based on the recommendations of an expert statistician and an expert in clinical education. A “very undesirable” environment indicates a very poor educational setting, where negative aspects outweigh positive aspects. An “undesirable” operation theater learning environment signifies that negative aspects are predominant over positive ones. A “moderately desirable” operation theater learning environment suggests numerous problems with the educational setting. In a “desirable” operation theater learning environment, positive aspects outweigh negative aspects. A “Very Desirable” environment denotes an excellent educational setting. The score is 160 [14]. The questionnaire was adapted from the Persian version of the Anesthetic Trainee Theater Educational Environment Measure (ATEEM) tool. It measures the learning environment for anesthesia students in the OR. The ATEEM tool consists of 40 items with a maximum possible score of 160. These materials are very undesirable (0–31), undesirable (32–63), moderately desirable (64–95), desirable (96–127) and very desirable (128–160).

### Data collection tool, methods and procedures

The data were collected using an English version of a self-administered structured questionnaire adapted from the Persian version of the Anesthetic Trainee Theater Educational Environment Measure (ATEEM) tool, which was designed to evaluate the learning environment of anesthesia students in the operating room (OR). The ATEEM tool comprises 40 items across five domains: autonomy, perceptions of the operation theater learning environment, workload support, perceptions of teachers and teaching-learning opportunities, and orientation to learning. The tool demonstrated strong internal consistency (Cronbach’s alpha of 0.95) and was validated for face and content validity by the developer. The questionnaire consists of two sections, with the first gathering demographic data such as sex, university name, and academic year. The second section included 40 items scored on a 5-point Likert scale (ranging from 0 to 4), with negatively worded questions reverse-coded. The maximum score for each domain is determined by multiplying the number of questions by the highest possible score for each item [14].

### Data quality management

To ensure data quality, we conducted pretesting of the data collection tool (the questionnaire) on a sample representing 5% of the student population. Completion of the questionnaire was promptly verified shortly after it was completed. Furthermore, before analysis, the obtained data were coded, cleaned, and investigated. Each data point was checked for completeness before being placed into the electronic data. The analysis did not include incomplete data.

### Data management and analysis

The data were entered into EpiData 4.6 and then exported to Statistical Software for Data Science (STATA) Version MP17 for cleaning and analysis. Descriptive statistics were used to describe participants’ sociodemographic status, while inferential analyses were performed to identify factors influencing the operation theater learning environment. The results are presented in the form of text, figures, tables, graphs, and charts. To determine the association between predictor variables and the outcome variable, the chi-square test was employed, and significant variables (with a *p* value less than 0.05 and a 95% confidence interval) were fitted for bivariable logistic analysis. Responses associated with the operating theater learning environment (with a *p* value less than or equal to 0.2) from the bivariable analysis were entered into generalized ordered logistic regression analyses to identify factors associated with the learning environment. A *p* value less than 0.05 in the 95% confidence interval was considered to indicate statistical significance. To address concerns about multicollinearity, the Spearman test, variance inflation factor (VIF), and tolerance test were used. All tolerance values greater than 0.1 and all VIF values less than 10 indicated that any significant relationships found were not inflated by correlations between the predictor variables. Adjusted odds ratios (AORs) with corresponding 95% confidence intervals (CIs) were calculated to determine the associations of the independent factors with the outcome variables. Box plots were used to check for outliers.

## Results

### Sociodemographic characteristics of the study participants

In this study, 313 undergraduate anesthesia students from 13 higher education teaching hospitals participated, for a response rate of 95.7%, as shown in Table 1.

**Table 1:** Sociodemographic characteristics of anesthesia students who learn at higher education teaching hospitals in Ethiopia, 2023

Variables	Frequency (n)	Percentage (%)	
**Sex**			
Male	163	52.08	
Female	150	47.92	
**Anesthesia program**			
Generic	256	81.79	
Post basic	57	18.21	
**Academic year**			
2nd year	39	12.46	
3rd year	81	25.68	
4th year	193	61.6	

### Anesthesia student’s perception of operation theater learning environments

**Fig. 1:**
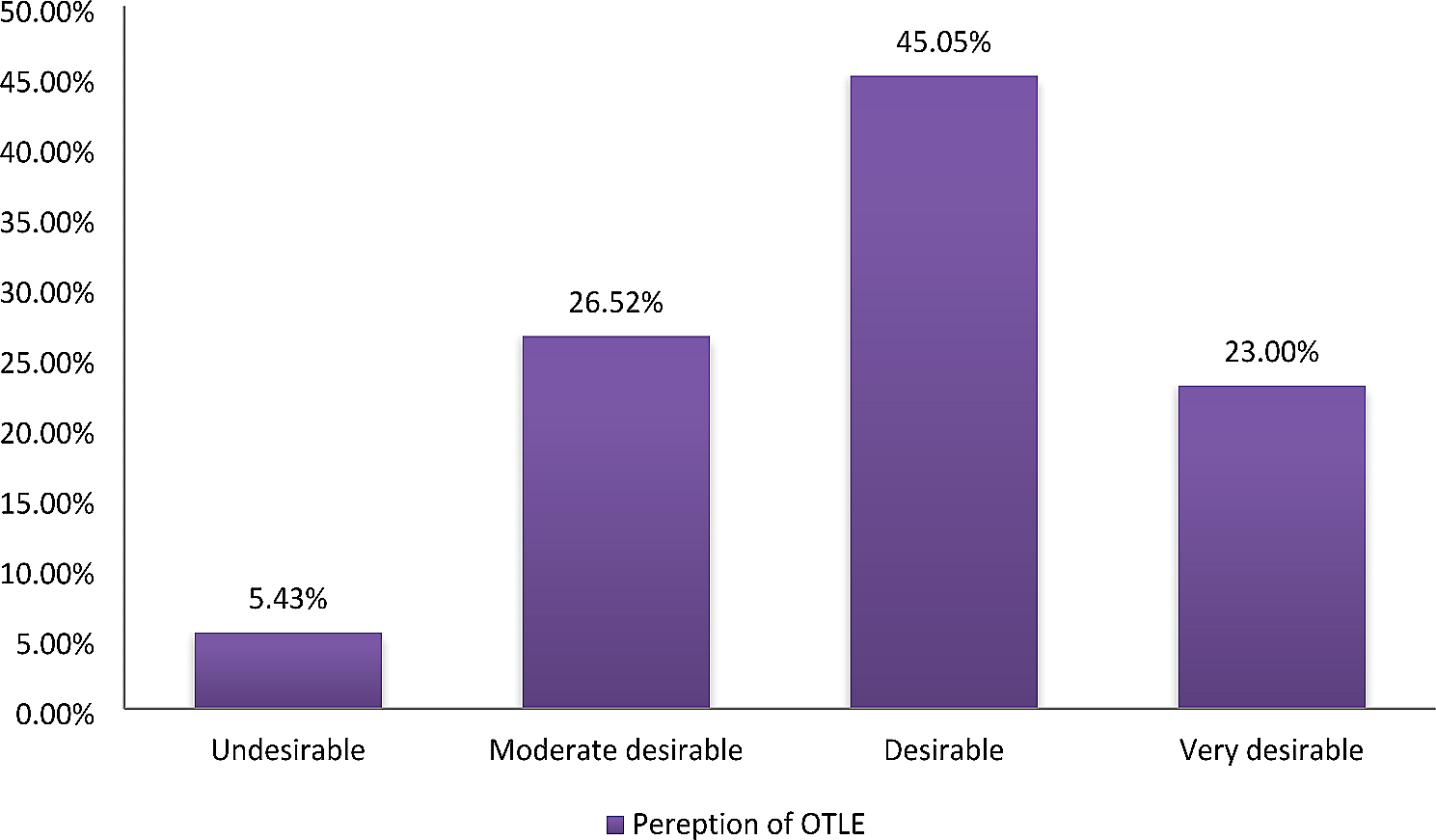
Bar graph of the outcomes of perception of the operating theater learning environment percentage in 2023

**Fig. 2:**
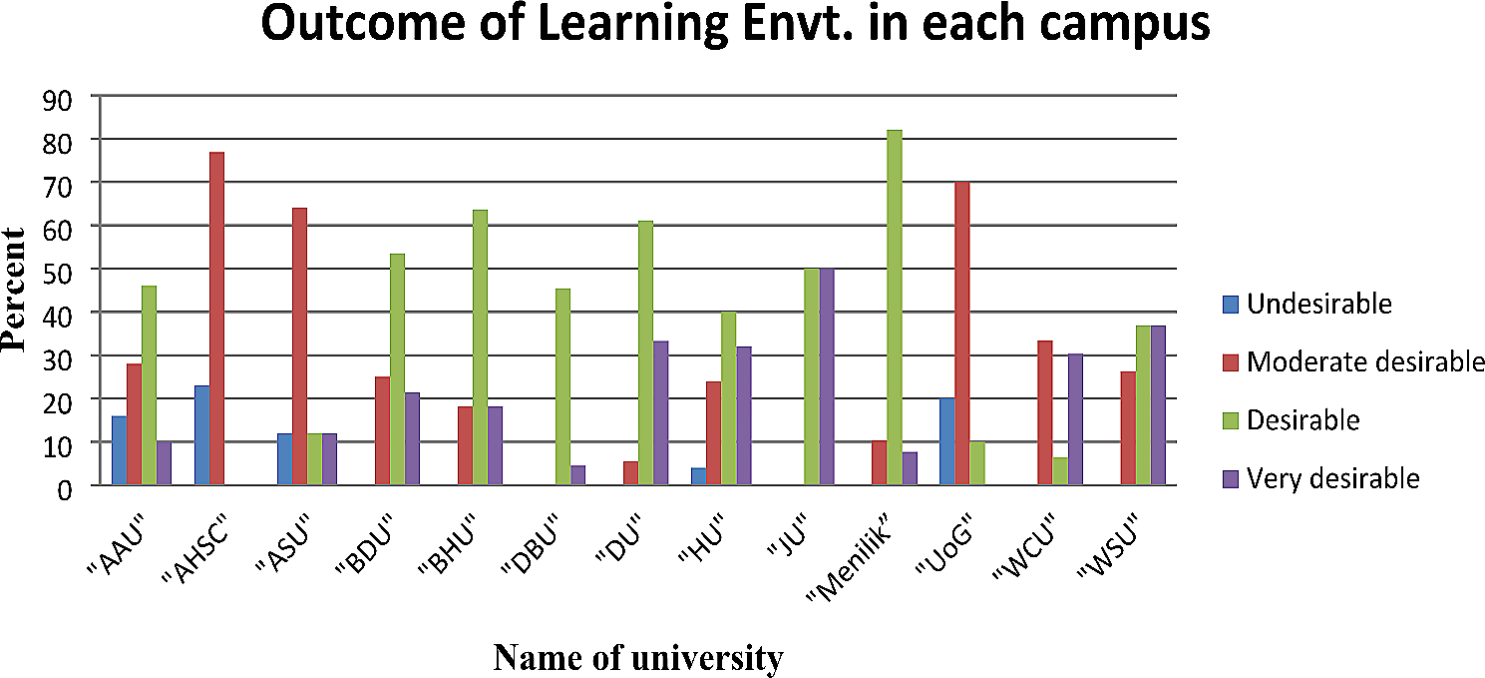
Bar graph of the outcomes of perceptions of the operating theater learning environment at each university in 2023

Nearly half of the participants 141(45.05%) rated the operation theater learning environment as desirable, while 83 (26.52%) rated it as moderately desirable. Approximately 72 (23%) of the participants rated it as very desirable, and only 17 (5.43%) considered it undesirable. Interestingly, none of the participants rated the environment as very undesirable in Ethiopian higher education teaching hospitals, as shown in Figs. 1 and 2.

### Factors affecting the perception of the operation theater learning environment and student responses

The study revealed that many students felt that operation theater placement did not adequately support learning 83(26.52%). A significant portion did not receive clinical demonstration 121(38.66%) or simulation 124(39.62%) before practicing on real patients. A greater number of students 236(75.4%) discussed elective surgical cases with their tutors preoperatively. Nearly half of the patients experienced fear during patient management 156(49.84%). Common issues included a lack of competent instructors 70(22.36%), a lack of teaching facilities (164, 52.4%), and insufficient number of theater sessions 85(22.16%) per week. Additionally, some students felt that their tutors lacked respect 96(30.67%) and were not effective role models 159(50.8%) (Table 2).

**Table 2:** Frequency of students’ responses to factors affecting their perception of the operation theater

Influencing factors	Frequency	Percentage
OR practice adequate Yes	230	73.48
No	83	26.52
**Reason of not adequate practice**		
Tutors miss OR program Yes	38	12.14
No	275	87.86
Inadequate learning resources Yes	60	20.13
No	250	79.87
Inadequate supervision Yes	54	17.25
No	259	82.75
Skill Lab before practicing on real patients Yes	192	61.34
No	121	38.66
Reason not practice in the Skill Lab		
Lack of demonstration doll Yes	59	18.85
No	254	81.15
Lack of skill lab Yes	60	19.17
No	253	80.83
Missing skill lab program Yes	67	21.41
No	246	78.59
Lack of skill lab technical assistant Yes	45	14.35
No	268	85.62
Clinical simulation practice Yes	189	60.38
No	124	39.62
Reason not practice with clinical simulation		
Lack of simulation equipment Yes	88	28.12
No	225	71.88
Lack of simulation learning room Yes	71	22.68
No	242	77.32
Lack of interest Yes	28	8.95
No	285	91.05
Discussion of elective cases preoperatively Yes	236	75.4
No	77	24.6
Reason not discuss cases preoperatively		
Absence of morning discussion trend Yes	52	16.61
No	261	83.39
Due to a concern of delay time for beginning time		
Yes	28	8.95
No	285	91.05
Missing of tutors from morning discussion Yes	36	11.5
No	277	88.5
Lack of morning discussion class room Yes	42	13.42
No	271	86.5
Behavior seen on student at clinical practice		
Lack of self-confidence Yes	117	37.38
No	196	62.62
Absenteeism from clinical area from time to time Yes	71	22.68
No	242	77.32
Overconfidence Yes	84	26.84
No	229	73.16
Fear during patient management Yes	156	49.84
No	157	50.16
Situations cause to fear students in the OR		
Clinical assessment Yes	30	9.58
No	258	90.42
Strict supervision Yes	47	15.02
No	266	84.98
Patient complication Yes	101	32.27
No	212	67.73
Death of patient Yes	53	16.93
No	260	83.07
Intrinsic factors make students fear in the OR		
Fear of making mistakes Yes	239	76.36
No	74	23.64
Lack of competency Yes	102	32.59
No	211	67.41
Fear of OR environment Yes	60	19.17
No	253	80.83
Measures will reduce students fear at the OR		
Appropriate clinical supervision Yes	210	67.09
No	103	32.91
Clinical case presentation Yes	118	37.7
No	195	62.3
Skill lab demonstration Yes	140	44.73
No	173	55.27
Resource is often missed in clinical practice		
Glove Yes	155	49.52
No	158	50.48
Syringe Yes	105	33.55
No	208	66.45
Drug Yes	140	44.73
No	173	55.73
Gown drape Yes	105	33.54
No	208	66.46
Common problem presents in OR environment		
Lack of competent clinical instructors Yes	70	22.36
No	243	77.64
Lack of teaching facilities in clinical area Yes	164	52.4
No	149	47.6
Inadequate number of patients Yes	97	30.99
No	216	69.01
Common factors in clinical area influence learning		
Non supportive environment Yes	97	30.99
No	216	69.01
Practicing under stressful situation Yes	173	55.27
No	140	44.73
Inadequate PPE (personal protective equipment Yes	104	33.23
No	209	66.77
Caring of serious patients Yes	73	23.32
No	240	76.68
Noise from music opened in the OR Yes	52	16.61
No	261	83.39
Enough theatre sessions per week Yes	228	72.84
No	85	27.16
Reason not enough theatre sessions per week		
Limited case flow to the hospital Yes	57	18.21
No	256	81.79
Number of student is large Yes	34	10.86
No	279	89.14
Inadequate number of OR table Yes	32	10.22
No	281	89.78
Lack of specific specialist Surgeon Yes	24	7.67
No	289	92.33
All clinical tutor respect their student Yes	217	69.33
No	96	30.67
All tutor role model for students Yes	154	49.2
No	159	50.8

### Factors associated with perceptions of the anesthesia operation theater learning environment

The study identified significant factors influencing the operation theatre (OR) learning environment through bivariable and multivariable analyses. A lack of teaching facilities reduced the odds of a very desirable learning environment by 84%, while the absence of tutors in clinical areas reduced the odds by 96.5%. Noise disturbance decreased odds by 80%, while students in generic anesthesia programs had 4.59 times greater odds of a very desirable environment. Tutors respecting students increased odds by 3.5 times, and being a role model increased odds by 3.2 times. Preoperative discussion of surgical cases with tutors increased the odds by 4.98 times, but strict supervision during patient management decreased the likelihood of a very desirable operation theatre learning environment by 75% (Table 3).

**Table 3:** Multivariable generalized ordered logistic model (STATA) output

**Generalized Ordered Logit Estimates**		Number of obs	=		313	
		LR χ^2 (51)	=		231.17	
		Prob > χ^2	=		0.0000	
Log likelihood = -262.3545		Pseudo R2	=		0.3058	
**Operation theater learning Envit.**	**Odds ratio**	**Std. err.**	**Z**	**P > z**	**[95% conf. interval]**	
**Undesirable**						
Program					**LB**	**UB**
Generic	1.45928	1.349657	0.41	0.683	.2381665	8.94122
Adequate OR practice						
Yes	7.09e-07	.0006478	-0.02	0.988	0	.
Tutors miss OR program						
Yes	.8065655	.9838598	-0.18	0.860	.0738469	8.809416
Inadequate resources						
Yes	2.13e-07	.0001946	-0.02	0.987	0	.
Appropriate supervision						
Yes	1.655731	2.233668	0.37	0.709	.1176725	23.29726
Skill Lab practice						
Yes	5.131318	6.807716	1.23	0.218	.3810136	69.10627
Missing skill lab practice						
Yes	.2235655	.2318123	-1.44	0.149	.0292958	1.706102
Clinical Simulation practice						
Yes	3.54229	3.60214	1.24	0.214	.4827259	25.99366
Lack of Simulation equipment						
Yes	5.174633	5.988509	1.42	0.156	.5355492	49.99882
Preop discussion with tutor						
Yes	.1834731	.161341	-1.93	0.054	.0327378	1.028241
Clinical assessment of students						
Yes	.5949412	.5540002	-0.56	0.577	.0959065	3.690625
Strict supervision of student at OR						
Yes	.2578725	.2100279	-1.66	0.096	.0522555	1.27256
Lack of teaching facilities at OR						
Yes	.1614326	.1264346	-2.33	**0.020**	.0347798	.7492997
Non supportive environment						
Yes	1.176395	.8423419	0.23	0.821	.2891093	4.786793
Noise from played music						
Yes	.1657353	.16442	-1.81	0.070	.023712	1.158407
All tutor Respect student						
Yes	.1990102	.1881504	-1.71	0.088	.0311977	1.269485
All tutor role model for student						
Yes	1.848682	1.688962	0.67	0.501	.308462	11.07956
_cons	5.62e + 08	5.13e + 11	0.02	0.982	0	.
**Moderate desirable**						
Program						
Generic	.575439	.284904	-1.12	0.264	.2180536	1.518572
Adequate OR practice						
Yes	.4423369	.4076495	-0.89	0.376	.072661	2.692805
Tutors miss OR program						
Yes	.0356111	.0336063	-3.53	**0.001**	.0056015	.2263944
Inadequate resources						
Yes	1.825763	1.443523	0.76	0.446	.3876591	8.598823
Appropriate supervision						
Yes	.2645681	.2237071	-1.57	0.116	.0504428	1.387637
Skill Lab practice						
Yes	1.829754	.8026776	1.38	0.168	.7744389	4.32313
Missing skill lab practice						
Yes	.5378372	.2964447	-1.13	0.260	.1825939	1.58422
Clinical Simulation practice						
Yes	2.272414	1.130809	1.65	0.099	.8568576	6.026514
Lack of Simulation equipment						
Yes	1.612261	.8987158	0.86	0.392	.5406967	4.807475
Preop discussion with tutor						
Yes	1.466369	.6169255	0.91	0.363	.6428786	3.344704
Clinical assessment of students						
Yes	.7080483	.4540051	-0.54	0.590	.2014963	2.488048
Strict supervision of student at OR						
Yes	.4451919	.2339557	-1.54	0.124	.1589377	1.247004
Lack of teaching facilities at OR						
Yes	1.099993	.3770523	0.28	0.781	.5618466	2.153586
Non supportive environment						
Yes	.9458613	.3681023	-0.14	0.886	.4411274	2.028107
Noise from played music						
Yes	.2203908	.118089	-2.82	**0.005**	.0771091	.629914
All tutor Respect student						
Yes	3.44236	1.307264	3.26	**0.001**	1.635331	7.246145
All tutor role model for student						
Yes	3.237349	1.244884	3.05	**0.002**	1.523597	6.87874
_cons	1.200278	1.34105	0.16	0.870	.1343535	10.72296
**Desirable**						
Program						
Generic	4.58661	3.224899	2.17	**0.030**	1.156117	18.19624
Adequate OR practice						
Yes	.0768046	.1151484	-1.71	0.087	.0040666	1.45058
Tutors miss OR program						
Yes	.3681437	.5387579	-0.68	0.495	.0209089	6.481919
Inadequate resources						
Yes	.0951133	.1383351	-1.62	0.106	.0054984	1.645314
Appropriate supervision						
Yes	.0643321	.1015227	-1.74	0.082	.0029184	1.418116
Skill Lab practice						
Yes	2.307784	1.015597	1.90	0.057	.9740991	5.467481
Missing skill lab practice						
Yes	1.10014	.7397554	0.14	0.887	.2944991	4.109721
Clinical Simulation practice						
Yes	1.838567	1.041355	1.08	0.282	.6058475	5.579504
Lack of Simulation equipment						
Yes	1.068715	.6884797	0.10	0.918	.3023477	3.777607
Preop discussion with tutor						
Yes	4.982886	3.385274	2.36	**0.018**	1.315805	18.86993
Clinical assessment of students						
Yes	1.032774	.7474108	0.04	0.964	.2500329	4.265927
Strict supervision of student						
Yes	.2495142	.1608815	-2.15	**0.031**	.0705113	.8829421
Lack of teaching facilities at OR						
Yes	.8881178	.2940168	-0.36	0.720	.4641679	1.699284
Non supportive environment						
Yes	.6552585	.2696581	-1.03	0.304	.2924949	1.467936
Noise from played music						
Yes	.5189199	.2987325	-1.14	0.254	.1679111	1.603693
All tutor Respect student						
Yes	3.432509	1.75948	2.41	**0.016**	1.256874	9.374142
All tutor role model for student						
Yes	1.978892	.6952529	1.94	0.052	.9939524	3.939839
_cons	.033666	.0577514	-1.98	**0.048**	.0011669	.9713156
**Very desirable**						

Note: _cons estimates baseline odds

## Discussion

The operating room is a unique and dynamic learning environment that caters to a wide range of learning styles, encompassing spatial, aural, verbal, physical, logical, interpersonal, and intrapersonal aspects [7]. In this study, 23% and 45.05% of participants perceived operation theater learning environments as very desirable and desirable, respectively. However, these figures are lower than those of similar studies conducted at the University of Witwatersrand (30.6%, 67.1%) and in Thailand (48.4% and 51.6%), respectively [3, 31].

Among all the participants, 83 (26.52%) and 17 (5.43%) stated that the operation theater learning environment was moderately desirable and undesirable, respectively. This finding contrasts with a study conducted in Thailand, which reported that only 2.4% and 0% of respondents reported a moderately desirable and undesirable learning environment, respectively [31]. The difference may be attributed to their use of a different cutoff point for the tool’s category levels. Their total score was 164, while this study scored out of 160 based on tool recommendations. Additionally, the use of more advanced medical equipment and technology in Thailand’s anesthesia operation theaters compared to the lack of teaching facilities in our country may have influenced these results, leading to no reported instances of an undesirable operation theater learning environment in Thailand.

This study highlights how the absence of teaching facilities in the operating room (OR) detrimentally impacts the creation of an optimal learning environment. This finding correlates with findings from the study “Educational Resources for Anesthesia Training Programs,” which concludes that teaching facilities in anesthesia education environments often fall short of meeting national standards [32]. The expansion of professional anesthesia training programs in low- and middle-income nations underscores the urgent need for investment in teaching materials. A sufficient supply of teaching instruments and resources is indispensable for fostering an efficient learning environment in the operating theatre. This study sheds light on a critical aspect of medical education: the importance of adequate teaching facilities in the operating room (OR) for creating an optimal learning environment. The finding that the absence of such facilities can detrimentally impact learning underscores the need for attention and investment in this area. It not only affects the quality of education for current students but also has broader implications for the future of healthcare.

The likelihood of experiencing a highly desirable learning environment in the operation theater decreased significantly by 96.5% when tutors were absent from the clinical learning area. This conclusion is reinforced by a survey conducted in the United States of America on the supervision of anesthesia trainees, which revealed that inadequate supervision can have a detrimental impact, reducing the learning environment by 90%. This deficiency not only affects student education but also compromises patient care and safety [33]. Students who reported higher levels of supervision performed procedures more frequently than did those with lower supervision scores. Insufficient supervision has been linked to a greater frequency of patient deaths under the care of junior students, thereby affecting the desirability of the operation theater learning environment. It is plausible that anesthesia training programs burdened with heavy clinical workloads struggle to provide trainees with the necessary supervision. This was further evidenced by the inverse correlation between low supervision and the assertion that excessive clinical workload poses a significant obstacle to effective supervision. Our findings suggest that academic institutions encounter greater challenges in delivering adequate supervision to trainees when faced with a greater demand for clinical services. the study’s findings highlight the multifaceted implications of inadequate supervision in anesthesia training. They underscore the importance of prioritizing supervision in medical education and call for concerted efforts to address the underlying factors contributing to this issue. By investing in supervision, institutions can create a more supportive learning environment, enhance procedural proficiency among trainees, and ultimately improve patient care and safety.

The likelihood of achieving a very desirable learning environment was significantly reduced by 80% when noise from playing music disrupted the operating room (OR). This discovery is supported by a systematic review conducted on noise and music in the operating room, which emphasizes the need for caution regarding music played in such settings. Questions arise regarding its safety concerning communication and distraction due to the elevated decibel levels it introduces [34]. Another study further illustrated the challenges posed by music-induced noise in effectively hearing, understanding, and communicating, thus impacting the desirability of the operation theater learning environment. Given that poor communication is a significant contributor to adverse events, addressing such distractions becomes imperative. The Environmental Protection Agency (EPA) recommends a maximum background noise level of 45 decibels (dB) in hospitals, yet even this level can prove distracting [35]. Monitoring noise levels during trauma procedures in operating rooms revealed an average noise level of 85 dB, ranging from 40 to 130 dB [36]. Notably, orthopedic and neurosurgical procedures exhibit some of the highest continuous background noise levels, with intermittent peak levels exceeding 100 dB occurring more than 40% of the time [35]. In a laboratory experiment simulating OR background noise, anesthesia students’ ability to accurately identify changes in saturation on a pulse oximeter decreased by 17% [37].

The discussion of elective surgical cases with a tutor before an operation significantly increased the likelihood of achieving a very desirable learning environment in the operation theater by 4.98 times. This finding is supported by a study conducted by Zhou Y, which concluded that preoperative discussions for students, facilitated through standardized training in the Department of Anesthesiology, were highly effective, practical, and tailored to individual needs [38]. During these discussions, the benefits of four learning approaches—problem-based, patient-focused, disease-centered, and learner-focused—are seamlessly integrated into interactive teaching sessions. This approach not only fosters active student engagement but also enhances clinical reasoning skills by encouraging participation in the analysis and resolution of clinical questions [38]. Topics covered in preoperative discussions may encompass various aspects, including preoperative evaluation, anesthetic considerations, anesthetic techniques, and conditions requiring heightened vigilance [6].

Being a role model for students increases the likelihood of creating a very desirable learning environment by 3.2 times. The findings of this study highlight the crucial role of clinical tutors in facilitating students’ socialization and the establishment of their professional identities as they embark on a career in anesthesia, consistent with the literature [39–42]. Students learn not only from formal instruction but also through the observation, imitation, and emulation of their teachers. Clinicians who serve as role models contribute significantly to students’ development of professional skills, values, and attitudes. Three key attributes define an effective role model: clinical skills, teaching abilities, and personal qualities [43]. The responsibility of the teacher is to exemplify and demonstrate various components of anesthesia practice, such as the psychomotor skills involved in endotracheal intubation or the judgment required for devising an anesthesia induction plan. Subsequently, the learner is encouraged to understand, practice, and apply these essential components repeatedly [6]. Teachers are responsible for exemplifying and demonstrating various components of anesthesia practice, such as psychomotor skills and judgment required for anesthesia induction plans. By consistently demonstrating these essential components, teachers encourage learners to understand, practice, and apply them repeatedly. This approach facilitates comprehensive skill acquisition and professional growth among students, ultimately enhancing the overall learning experience in anesthesia education.

The likelihood of achieving a very desirable learning environment increases by 3.5 times when all tutors respect anesthesia students. Within the operating room (OR), it is imperative for clinical educators to regard students with respect as individuals to facilitate effective learning. A study assessing various factors influencing learning experience revealed that the level of respect shown by clinical instructors toward anesthesia students had the strongest correlation with the creation of a very desirable learning environment, as ranked by students among 27 different factors [44]. Respecting anesthesia students not only fosters greater interaction between tutors and students but also plays a pivotal role in shaping a supportive and conducive atmosphere for learning within the OR. This culture of respect not only enhances the educational experience but also nurtures professional growth and development among students.

“Strict supervision of students during patient management in the operating room (OR) reduces the likelihood of creating a very desirable learning environment by 75%. This finding is supported by a study conducted at the Mayo Clinic, which indicated that strict supervision can significantly impede students’ desire to learn in the OR by impacting their decision-making abilities and confidence [45]. Anesthesia students may independently write orders for diagnostic studies and therapeutic interventions. The decision to adjust the instructor-to-student ratio from 1:1 to 1:2 or to allow instructors to leave the OR for periods of time depends on several factors: the student’s knowledge and ability, the patient’s physical status, the complexity of the anesthesia and/or surgical procedure, and the instructor’s experience [46]. The term “supervision” refers to a variety of activities, including being physically present during critical moments of a case, participating in anesthesia planning, providing clinical and educational guidance throughout the anesthetic, and granting autonomy to supervised individuals with feedback [45]. Future research in anesthesia education and training should focus on several key areas to enhance the learning environment and promote patient safety. This includes investigating the impact of specific teaching facilities and resources on student learning outcomes, optimal supervision practices in terms of instructor-to-student ratios and feedback methods, strategies for managing noise levels in the operating room, and the refinement of preoperative discussions between tutors and students.

### Strengths and limitations of the study

Comprehensive data collection from primary sources across all Ethiopian higher education institution teaching hospitals contributes to the generalizability of the findings to the national population of anesthesia students in Ethiopian teaching hospitals. The potential for future research to utilize this study as a benchmark for assessing the learning environment of anesthetic operation theaters underscores its significance. The inherent limitations of cross-sectional studies include the inability to establish cause-and-effect relationships. There are possible biases in the data collection methods. Potential for confounding variables not accounted for in the study.

## Conclusion

In conclusion, the findings of this study revealed that the highest frequency of perception regarding the operation theater learning environment in Ethiopia was deemed desirable by 141 participants (45.05%), with no study participants indicating that OTLEs in operation theaters were very undesirable. Factors strongly associated with the OTLE in the OR for anesthesia students include the lack of teaching facilities in the OR, the absence of tutors from the OR, noise from music played in the OR, tutors’ respect for their students, tutors serving as role models for their students, preoperative discussions with tutors, and the strict supervision of students.

### Recommendation

The strict supervision of anesthesia students significantly affects the desirability of the operation theater environment. Therefore, instructor supervision should be tailored based on the student’s knowledge and ability, the patient’s physical status, the complexity of the anesthetic and/or surgical procedure, and the instructor’s experience. It is essential for all tutors to serve as role models for their students whenever possible. The three defining characteristics of a good role model are clinical skills, teaching abilities, and personal qualities. Therefore, efforts should be made to ensure that tutors exhibit these qualities in their interactions with students. Universities and anesthesia departments need to acknowledge the demanding nature of anesthesia operation theater practices. Consequently, they should collaborate to provide the necessary resources and teaching facilities to support effective teaching and learning in this environment. It may be beneficial to conduct additional studies that include managers and instructors as study participants. This would provide valuable insights into their perspectives on the operation theater learning environment and potential strategies for improvement.

### Electronic supplementary material

Below is the link to the electronic supplementary material.

Supplementary Material 1

## Data Availability

The data sets used and analyzed during the study are available from the corresponding author upon reasonable request.

## References

[1] Kundra P, Kurdi M, Mehrotra S, Jahan N, Kiran S, Vadhanan P (2022). Newer teaching-learning methods and assessment modules in anaesthesia education. Indian J Anaesth.

[2] Wacker J, Staender S (2014). The role of the anesthesiologist in perioperative patient safety. Curr Opin Anaesthesiol.

[3] Khan S, Scribante J, Perrie H, Green-Thompson L (2021). Evaluation of the anaesthetic theatre educational environment at the University of the Witwatersrand. South Afr J Anaesth Analgesia.

[4] Gemuhay HM, Kalolo A, Mirisho R, Chipwaza B, Nyangena E. Factors affecting performance in clinical practice among preservice diploma nursing students in Northern Tanzania. Nursing Research and Practice. 2019;2019. 10.1155/2019/3453085 PMC6420979 30941212

[5] Nordquist J, Hall J, Caverzagie K, Snell L, Chan M-K, Thoma B (2019). The clinical learning environment. Med Teach.

[6] Viola L, Young DA (2016). How to teach anesthesia in the operating room. Int Anesthesiol Clin.

[7] Bakhshialiabad H, Bakhshi M, Hassanshahi G. Students’ perceptions of the academic learning environment in seven medical sciences courses based on DREEM. Advances in medical education and practice. 2015:195–203. 10.2147/AMEP.S60570 PMC4376065 25848331

[8] Young JQ, Van Merrienboer J, Durning S, Ten Cate O (2014). Cognitive load theory: implications for medical education: AMEE Guide 86. Med Teach.

[9] Hunukumbure AD, Leedham-Green KE, Rajamanoharan A, Patel K, Tang A, Das S (2022). Twelve tips for surgeons to maximise medical student learning in the operating theatre. Med Teach.

[10] Pattni N, Arzola C, Malavade A, Varmani S, Krimus L, Friedman Z (2019). Challenging authority and speaking up in the operating room environment: a narrative synthesis. Br J Anaesth.

[11] Rayatdoost E, Jahromi RR, Ayalbar A, Kalani N (2022). Factors affecting the quality of Clinical Education from the perspective of medical students. Int J Med Invest.

[12] Witteles RM, Verghese A (2016). Accreditation Council for Graduate Medical Education (ACGME) milestones—time for a revolt?. JAMA Intern Med.

[13] Owei L, Neylan CJ, Rao R, Caskey RC, Morris JB, Sensenig R (2017). In situ operating room-based Simulation: a review. J Surg Educ.

[14] Koohpayehzadeh J, Mirzaei Z, Zahedi H, Alebouyeh MR, Naghizadeh Moogari Z (2019). Psychometric properties of the Persian version of the anesthetic Trainee Theatre Educational Environment measure (ATEEM). Med J Islam Repub Iran.

[15] Jones R, Morris R (2016). Facilitating learning in the operating theatre and intensive care unit. Anaesth Intensive Care.

[16] Gaberson K, Oermann M, Gaberson KB, Offermann MH, Shellenbarger T (2015). Contextual factors affecting clinical teaching. Clinical teaching strategies in nursing.

[17] McSharry E, Lathlean J (2017). Clinical teaching and learning within a preceptorship model in an acute care hospital in Ireland; a qualitative study. Nurse Educ Today.

[18] Ravindra P, Fitzgerald JEF, Bhangu A, Maxwell-Armstrong CA (2013). Quantifying factors influencing operating theater teaching, participation, and learning opportunities for medical students in surgery. J Surg Educ.

[19] Cordovani L, Cordovani D, Wong A (2022). Characteristics of good clinical teachers in anesthesiology from medical students’ perspective: a qualitative descriptive study. Can J Anesthesia/Journal Canadien d’anesthésie.

[20] Lu J, Bai J, Liu Z, Chi S, Yu Z, Shen L (2021). Application of CBL Teaching Method Combined with situational Simulation Teaching in Clinical Teaching of Anesthesiology and its influence on improving theoretical knowledge and clinical practice ability of Anesthesiology Medical Students. Tob Regul Sci.

[21] Anand S, Hashia AM, Thakur R (2022). Anaesthesia education of our times. Indian J Anaesth.

[22] Waseem T, Munir Baig H, Yasmin R, Ahmad Khan R (2020). Exploring operating room-based student learning experience: Perils & pitfalls? A narrative literature review. Archives Surg Res.

[23] Jeffree RL, Clarke RM (2010). Ten tips for teaching in the theatre tearoom: shifting the focus from teaching to learning. World J Surg.

[24] Zundel S, Wolf I, Christen H-J, Huwendiek S (2015). What supports students’ education in the operating room? A focus group study including students’ and surgeons’ views. Am J Surg.

[25] Sullivan SA, Anderson BM, Pugh CM (2015). Development of technical skills: education, simulation, and maintenance of certification. J Craniofac Surg.

[26] Champagne BJ (2013). Effective teaching and feedback strategies in the or and beyond. Clin Colon Rectal Surg.

[27] Croghan SM, Phillips C, Howson W (2019). The operating theatre as a classroom: a literature review of medical student learning in the theatre environment. Int J Med Educ.

[28] Bowrey DJ, Kidd JM (2014). How do early emotional experiences in the operating theatre influence medical student learning in this environment?. Teach Learn Med.

[29] Böckers A, Mayer C, Böckers TM (2014). Does learning in clinical context in anatomical sciences improve examination results, learning motivation, or learning orientation?. Anat Sci Educ.

[30] Smith NA, Castanelli DJ (2015). Measuring the clinical learning environment in anaesthesia. Anaesth Intensive Care.

[31] Vongspanich W, Komonhirun R, Srilumyai S (2020). Anesthesiology residents’ perception towards educational environment using ATEEM in a medical school in Thailand. Res Dev Med Educ.

[32] Yetneberk T, Woldegerima Y, Getnet H, Mollalign M, Firde M, Moore JN (2021). Educational Resources for Preservice Anesthesia Training Programs in Amhara Region, Ethiopia. Adv Med Educ Pract.

[33] De Oliveira GS, Rahmani R, Fitzgerald PC, Chang R, McCarthy RJ (2013). The association between frequency of self-reported medical errors and anesthesia trainee supervision: a survey of United States anesthesiology residents-in-training. Anesth Analg.

[34] Fu VX, Oomens P, Merkus N, Jeekel J (2021). The perception and attitude toward noise and music in the operating room: a systematic review. J Surg Res.

[35] Nurses AopR. Association of periOperative Registered Nurses-AORN. 2013.

[36] Pereira BMT, Pereira AMT, Correia CdS, Marttos AC, Fiorelli RKA, Fraga GP (2013). Interruptions and distractions in the trauma operating room: understanding the threat of human error. Revista do Colégio Brasileiro De Cirurgiões.

[37] Stevenson RA, Schlesinger JJ, Wallace MT (2013). Effects of divided attention and operating room noise on perception of pulse oximeter pitch changes: a laboratory study. Anesthesiology.

[38] ZHOU Y, LIU Y, LIU J. Requirements of anesthesiology bedside teaching and application of interactive teaching mode in resident standardized training. Chin J Med Educ Res. 2019:195–7.

[39] Crosby RH, Joy (2013). AMEE Guide 20: the good teacher is more than a lecturer-the twelve roles of the teacher. Med Teach.

[40] Hesketh E, Bagnall G, Buckley E, Friedman M, Goodall E, Harden R (2014). A framework for developing excellence as a clinical educator. Med Educ.

[41] Sutkin G, Wagner E, Harris I, Schiffer R (2018). What makes a good clinical teacher in medicine? A review of the literature. Acad Med.

[42] Hatem CJ, Searle NS, Gunderman R, Krane NK, Perkowski L, Schutze GE (2013). The educational attributes and responsibilities of effective medical educators. Acad Med.

[43] Burgess A, Goulston K, Oates K (2015). Role modelling of clinical tutors: a focus group study among medical students. BMC Med Educ.

[44] Hexter AT, O’Dowd-Booth C, Hunter A (2019). Factors that influence medical student learning in the operating room. Med Teach.

[45] Dexter F, Logvinov II, Brull SJ (2013). Anesthesiology residents’ and nurse anesthetists’ perceptions of effective clinical faculty supervision by anesthesiologists. Anesth Analgesia.

[46] Solomon-Rice P, Robinson N (2015). Clinical supervision and the use of a three-tiered hierarchical approach to evaluate student clinician performance. Perspect Adm Superv.

