# Supplementary material for: Perception of the operation theater learning environment and related factors among anesthesia students in Ethiopian higher education teaching hospitals: a multicenter cross-sectional study
Source: BMC Med Educ. 2024 Mar 19;24:303. doi: 10.1186/s12909-024-05320-6 (PMC10949678; doi:10.1186/s12909-024-05320-6)
Supplement: Supplementary file 1 — Supplementary Material 1 [file 12909_2024_5320_MOESM1_ESM.docx]

**Supplementary Table 1: Socio-demographic profile of anesthesia student**

| No. | Question. | Response | |
| --- | --- | --- | --- |
| 101 | Name of your University | _______________ | |
| 102 | Sex | Male | Female |
| 103 | Which Undergraduate Anesthesia program are you study | Generic | Post basic |
| 104 | Year of study | __________________ | |

**Supplementary Table 2: Factors influencing clinical learning environment questionnaire.**

| No. | Question. | Response |  |
| --- | --- | --- | --- |
| 201 | Does the clinical placement offer the opportunity to learn? | 1. Yes 2. No | |
| 202 | If your answer is no to the above question, why does clinical placement not give opportunities for learning? | 1. Absence of tutor 2. Inadequate learning resources 3. Inadequate supervision 4. Other…………….. | |
| 203 | Is there a clinical demonstration in the Skill Lab before practicing on actual patients? | 1. Yes 2. No | |
| 204 | If your answer to the above question is NO, why do not you practice in the Skill Lab? | 1. Lack of demonstration doll 2. Lack of skill lab 3. Missing of skill lab program 4. Lack of skill lab technical assistant. 5. Other…………… | |
| 205 | Is there a clinical simulation that is learned before practicing on real patients? | 1. Yes 2. No | |
| 206 | If you answer no to the above question, why not practice with clinical simulation? | 1. Lack of simulation equipment 2. Lack of simulation learning room 3. Lack of interest 4. Other…….. | |
| 207 | Would you discuss elective surgical cases with your tutor preoperatively? | 1. Yes 2. No | |
| 208 | If your answer to the above question is NO, then why do not discuss preoperative with your tutor? | 1. Absence of morning discussion trend 2. Due to a concern of delay time for beginning time of first case inscion time 3. Missing of tutors from morning discussion 4. Lack of morning discussion class room 5. Other ………….. | |
| 209 | What behaviors are you seeing on you at the clinical practice? | 1. Lack of self confidence 2. Absenteeism from clinical area from time to time 3. Unsafe practice 4. Overconfidence 5. Neither 6. Other…………. | |
| 210 | Did you become fear at the OR during patient management? | 1. Yes 2. No | |
| 211 | If your answer is yes, what situations cause to fear you in the OR? | 1. Clinical assessment 2. Strict supervision 3. Patient complication 4. Death of patient 5. Other________ | |
| 212 | Which Intrinsic factors make you fear in the OR? | 1. Fear of making mistakes 2. Lack of competency 3. Fear of OR environment 4. Other________ | |
| 213 | Which measures will reduce your fear at the OR? | 1. Appropriate clinical supervision 2. Clinical case presentation 3. Skill lab demonstration 4. Other______ | |
| 214 | Which resource is often missed in clinical practice? | 1. Glove 2. Syringe 3. Drug 4. Gown drape 5. Other______ | |
| 215 | Which of the following problem are present in your clinical learning environment? | 1. Lack of competent clinical instructors, tutors and Coordinators 2. Lack of teaching facilities in clinical area 3. Inadequate number of patients. 4. Other…………………………. | |
| 216 | Which factors in your clinical area influence clinical learning? | 1. Non supportive environment due to many patients 2. Practicing under stressful situation 3. Inadequate PPE (personal protective equipment) 4. Caring of serious patients 5. Noise from music opened in the OR 6. Other………… | |
| 217 | Did you get enough theatre sessions per week to gain the appropriate experience? | 1. Yes 2. No | |
| 218 | If your answer is NO for the above question what is the possible reason? | A. Limited case flow to the hospital  B. Number of student is large compared to the hospital capacity  C. Inadequate number of OR table  D. Lack of specific specialist Surgeon.  E. Other_________ | |
| 219 | Did all your clinical tutor have respect for his her students | 1. Yes 2. No | |
| 220 | Did all your tutor role model for you? | 1. Yes 2. No | |

**Supplementary Table 3: Assessment tool**

**Please indicate whether you: Strongly Agree (SA), Agree (A), Unsure (U), Disagree (D), Strongly Disagree (SD) with the following statements as it apply to Your own feelings about theatre teaching in your University teaching hospital. Please click the appropriate response**.

| **Anesthetic Theatre Education Environment Measure (ATEEM) Assessment tool.** | | | | | | |
| --- | --- | --- | --- | --- | --- | --- |
| No. | Statement | Level of Agreement | | | | |
|  |  | SA | A | U | DA | SD |
| 301 | There are opportunities for learning all desired clinical skills |  |  |  |  |  |
| 302 | The teaching helps to develop my confidence |  |  |  |  |  |
| 303 | I receive effective supervision from the clinical teachers |  |  |  |  |  |
| 304 | Surgeons do not like the noise of theatre teaching* |  |  |  |  |  |
| 305 | Teaching is done at appropriate times not affecting vigilance |  |  |  |  |  |
| 306 | I receive theatre teaching in an anesthetic specialty areas targeted at my learning needs |  |  |  |  |  |
| 307 | The teacher helps to develop my competence |  |  |  |  |  |
| 308 | My clinical teachers are accessible for advice |  |  |  |  |  |
| 309 | I experience friendly relations with my teachers in theatre |  |  |  |  |  |
| 310 | I am aware of my an anesthetic role in theatre |  |  |  |  |  |
| 311 | I have opportunities to learn and practice a variety of clinical |  |  |  |  |  |
| 312 | The clinical teachers in this hospital interact well with student |  |  |  |  |  |
| 313 | There is an informative an anesthetic trainee handbook |  |  |  |  |  |
| 314 | The people I work with are friendly |  |  |  |  |  |
| 315 | I feel responsible and accountable for the care given to my patients |  |  |  |  |  |
| 316 | I am able to acquire adequate technical skills in this post |  |  |  |  |  |
| 317 | My clinical teachers are fair in their evaluations |  |  |  |  |  |
| 318 | At this hospital I have access to help from more experienced  Colleagues |  |  |  |  |  |
| 319 | My clinical teachers promote an atmosphere of mutual respect |  |  |  |  |  |
| 320 | I have an appropriate level of clinical responsibility |  |  |  |  |  |
| 321 | There are good opportunities for trainees who fail to complete their training satisfactorily |  |  |  |  |  |
| 322 | My clinical teachers are clear in their teaching |  |  |  |  |  |
| 323 | Whenever I should participate in formal educational  programmes I get relief from theatre duties |  |  |  |  |  |
| 328 | There is sex discrimination in this post* |  |  |  |  |  |
| 325 | I am clear about the learning objectives of the theatre teaching session |  |  |  |  |  |
| 326 | There is a clinical training programmers here that allows me to get first-hand experience in a range of procedures. |  |  |  |  |  |
| 327 | I receive the necessary clinical supervision |  |  |  |  |  |
| 328 | I feel part of a team working here |  |  |  |  |  |
| 329 | I discuss the an anesthetic plan of cases with the theatre teacher |  |  |  |  |  |
| 330 | I have the opportunity to acquire the appropriate practical  Procedures for my level of training (e.g. intubation/  spinal anesthesia) |  |  |  |  |  |
| 331 | My workload in this job is fine |  |  |  |  |  |
| 332 | I have good collaboration with theatre staff |  |  |  |  |  |
| 333 | I am encouraged to visit patients pre-operatively |  |  |  |  |  |
| 334 | I have the opportunity for on the job learning |  |  |  |  |  |
| 335 | My clinical teachers have established good rapport with me |  |  |  |  |  |
| 336 | I am encouraged to participate in the theatre setting |  |  |  |  |  |
| 337 | There is a systematic clinical training programme |  |  |  |  |  |
| 338 | I feel able to ask the questions I want |  |  |  |  |  |
| 339 | Much of what I learn seems relevant to my career |  |  |  |  |  |
| 340 | I feel comfortable in theatre socially |  |  |  |  |  |
